# Vision-Based People Detection System for Heavy Machine Applications

**DOI:** 10.3390/s16010128

**Published:** 2016-01-20

**Authors:** Vincent Fremont, Manh Tuan Bui, Djamal Boukerroui, Pierrick Letort

**Affiliations:** 1 Sorbonne Universités, Université de Technologie de Compiègne, CNRS, UMR 7253, Heudiasyc-CS 60 319, 60 203 Compiègne Cedex, France; manhtuanbui7687@gmail.com (M.T.B.); Djamal.Boukerroui@hds.utc.fr (D.B.); 2 Technical Center for the Mechanical Industry (CETIM), 60300 Senlis, France; Pierrick.Letort@cetim.fr

**Keywords:** heavy machines, sensor fusion, pedestrian detection, deformable part model, fisheye images, histogram of oriented gradients

## Abstract

This paper presents a vision-based people detection system for improving safety in heavy machines. We propose a perception system composed of a monocular fisheye camera and a LiDAR. Fisheye cameras have the advantage of a wide field-of-view, but the strong distortions that they create must be handled at the detection stage. Since people detection in fisheye images has not been well studied, we focus on investigating and quantifying the impact that strong radial distortions have on the appearance of people, and we propose approaches for handling this specificity, adapted from state-of-the-art people detection approaches. These adaptive approaches nevertheless have the drawback of high computational cost and complexity. Consequently, we also present a framework for harnessing the LiDAR modality in order to enhance the detection algorithm for different camera positions. A sequential LiDAR-based fusion architecture is used, which addresses directly the problem of reducing false detections and computational cost in an exclusively vision-based system. A heavy machine dataset was built, and different experiments were carried out to evaluate the performance of the system. The results are promising, in terms of both processing speed and performance.

## 1. Introduction

**Figure 1:**
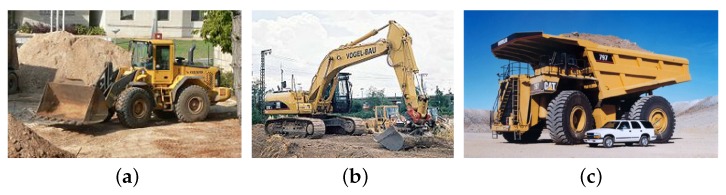
Common types of heavy machines. (**a**) Loader; (**b**) excavator; (**c**) truck.

Construction sites are always considered a high-risk working environment. People who work near heavy machines are constantly at risk of being struck by machines or their components. Accidents between machines and people represent a significant proportion of health and safety hazards in construction. Well-trained operators and protective equipment can reduce injuries and deaths, but it is difficult to eliminate these hazards completely. In many cases, accidents are caused by operators who are experienced, but who encounter problems of visibility, especially when using large vehicles. Drivers must keep watching all around their vehicles while performing a productive task. The most experienced and watchful driver may not notice people working in the vicinity of a machine, especially in blind angles. Without the help of effective detection devices, safety is very difficult to maintain. Because of the wide variety of types of heavy machine (see Figure 1), the accidents they cause can be of a number of different kinds.

The National Institute for Occupational Safety and Health (NIOSH) published a report on safety hazards in construction sites in the USA [1]. During the period 2003–2010, 962 people were killed on construction sites; 87% of these deaths were of people working on site at the time of the accident, and the remaining 13% were of people who simply happened to be on the site. The most common cause of death was being struck by moving equipment, followed by falling from moving equipment and being inside machines that collided with other machines. Accidents caused by moving equipment accounted for 76% of fatal injuries.

### 1.1. People Detection Systems

In view of these worrying statistics, efforts have been made to improve the safety for people working around heavy machines. Measures taken have a social impact, in terms of the casualties that are avoided and the costs that are incurred. Importantly, innovations and developments linked to these measures are also interesting from a scientific perspective. In the literature, there are three radically different approaches: infrastructure design, passive safety systems and active safety systems. The first of these, the infrastructure design approach [2], is suitable for large construction projects (large buildings, roads, bridges, *etc.*), but not suitable for small-scale everyday operations, such as maintenance, trucks moving outside construction sites, road resurfacing, street repairs, *etc*. The second approach, passive safety systems, has led to known improvements. This research direction is often known as improving safety through design, and an example of this would be protecting a pedestrian’s head in the case of a crash [3]. This kind of approach has been popular and has shown promising results for automobiles, but applying it to heavy machines has met various practical problems as a consequence of their various sizes and complicated shapes. We might also remark that the passive approach can be useful in reducing the severity of an accident, but it is not a radical solution.

The third approach, active safety systems, considers that prevention is better than cure, and active safety solutions now represent the principal area of research into safety improvements in heavy machines. Research has moved toward intelligent systems that are able to predict dangerous situations and anticipate accidents. These intelligent systems are referred to as advanced driver assistance systems (ADASs), in the sense that they help the driver by providing warnings and assistance in decision making, and they can even take automatic evasive actions in extreme cases.

Pedestrian detection systems (PDSs) are a particular type of ADAS. A PDS is capable of detecting both stationary and moving people in the vicinity of the vehicle in order to provide information to the driver. In some cases, it can perform overriding braking actions if needed. Visual pedestrian detection is a research area that goes back more than a decade [4]. Most of the techniques and methods have come from the fields of robotics, computer vision and machine learning, and they have received much attention because of their applicability to industrial safety systems, especially in the context of ADASs.

Although the issues are largely similar, there are certain characteristics and requirements specific to the context of heavy machines. Unlike a car, which needs to stop if there is an obstacle irrespective of whether that obstacle is a pedestrian or another object, a heavy machine needs to be able to identify a human as such, wherever the main requirement is human safety. Besides, a car often operates at a higher speed. While the system embedded in a car needs to be able to detect people at some distance, heavy machines need a wider field-of-view (FOV) to cover the area in close proximity. Construction machines are often large and have a complex shape, which also makes a wide FOV desirable.

For a car, it is reasonable to assume that a dominant plane exists, because most roads are locally flat. Such a hypothesis is important for detecting obstacles and regions of interest (ROI) in view frames. This hypothesis is, however, not always true for heavy machine environments, and this represents an essential difference between the two applications.

### 1.2. Structure and Contributions of This Paper

This article is a synthesis of previously-published conference papers with a new review of the relevant state-of-the-art, new theoretical developments and extended experimental results. The individual contributions have never been published together, and the aim of this paper is to show the global scope of vision-based perception systems that have been proposed for detecting people in heavy machine applications. In [5], a comparative study was conducted to show how to handle image distortions by enriching the training dataset with simulated sample deformation. An adaptive deformable part model (DPM) was proposed in [6] for directly adjusting image distortions in a fisheye context during the detection process. Finally, a multi-sensor system consisting of a fisheye camera and a LiDAR was developed to increase detection robustness and to handle different camera positions [7]. The main additional contributions of the present paper are as follows. In the case of enriching the training dataset with simulated sample deformation, a comparative study of several padding border functions is evaluated. A new theoretical development of the adaptive-DPM approach is presented. An extended evaluation is made of the performances of the proposed system with two different positions of the fisheye camera, using a new multi-sensor dataset for people detection in heavy machine applications with new scenarios. The paper is organized as follows. Section 2 presents the proposed perception system, the experimental dataset and the evaluation method. Works relating to visual people detection are then described in Section 3. In Section 4, the distortion analysis for fisheye images is presented. In Section 5, the detection approach using a mixed training dataset is described. In Section 6, the adaptive deformable part model is detailed. In Section 7, an extension to multi-sensor people detection is proposed. Finally, Section 8 concludes the paper and presents some future works.

## 2. Proposed Perception System for People Detection

### 2.1. Proposed Combination of Sensors for Heavy Machine Applications

PDSs can be based on active or passive sensors. As part of intelligent vehicle research, various kinds of sensors, including ultrasonic sensors, LiDARs, radars, RFIDs and cameras, have been tested and compared, individually [8,9] or in combination [10,11,12]. Different types of sensor have different advantages and drawbacks. For example, active range sensors (LiDAR, radar, ultrasonic) are very good at estimating distances in real time, while cameras not only have the advantage of a high resolution (horizontal and vertical), but also provide other useful information, like texture, color and depth. Range sensors are often used in obstacle detection applications. They are often present in heavy machines, but are not very practical, given that heavy machines often work close together and have interactions with different kinds of obstacles. Compared to other perception sensors, such as GPS, inertial or distance sensors (radar, LiDAR, ultrasonic), cameras provide the most information and, because of their versatility, give both high-level contextual and low-level geometrical information about the observed scene, at a high speed and low cost. Cameras are passive sensors that consume little energy and are easily miniaturized. In this paper, the focus is on the recognition of human figures, so cameras are the obvious choice, and because of the application and the requirement of a wide field-of-view, the fisheye camera will be used as the main sensor. Indeed, most heavy machines have strong morphological constraints (size, articulated body and machine end-tools’ position), and sensors can only be installed in limited specific areas to avoid shocks while providing a large field of view within a limited space. Compared to multi-camera systems, like the Blaxtair system from Arcure company (Paris, France) [13], fisheye cameras introduce strong distortions, but offer a wider field of view with limited cost within a smaller area. Moreover, since many heavy machines are already equipped with range sensors, like dedicated ultrasonic sensors, the need for depth information is not always justified. Through this paper, the extension from fisheye camera-based to omnidirectional camera-based object detection is straightforward, and in that case, distortions must also be considered within the recognition procedure.

However, the use of cameras is not entirely straightforward and poses a number of technological and theoretical problems relating to how they perceive their environment. The main drawback of camera technology is its low reliability in bad light conditions. For now, most vision systems applied to the safety of construction machines do not have integrated recognition functions and are often used with a radar as an assistance [14].

However, range information is valuable and can be obtained in different ways. By means of vision, it is possible to use time-of-flight (ToF) cameras [15] or a stereo vision system [10]. ToF cameras constitute a new sensor technology that is currently inappropriate for heavy machine applications, mainly because of their actual low resolution, range limitation and cost compared to new solid state LiDAR sensors, like the one from Quanergy (Sunnyvale, CA, USA) [16]. Stereo vision systems also have drawbacks, including a high computational cost for the disparity map and a limited detection area due to the necessity of cameras overlapping.

Because dense range information is not essential, range sensors, including ultrasonic sensors, LiDARs and radars, are more suitable. In practice, the range sensors most often found in heavy machines are ultrasonic, but in this work, we employ a single-layer LiDAR in order to utilize the precise measurements given by the LiDAR and so to avoid the problem of uncertainty in determining the positions of obstacles. LiDAR sensors are also compact, and the processing load is lighter than for ultrasonic sensors.

**Figure 2:**
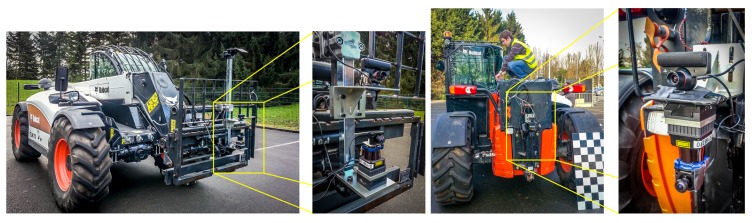
The telescopic forklift Bobcat TL470: Real images of the acquisition system setup in two different configurations.

For the experimental platform, the perception system consists of one fisheye camera (Point Grey Firefly MV USB2.0), one conventional camera (Sony PlayStation Eye for PS3) and one range-sensor (LiDAR Hokuyo UTM-30LX-EW). The sampling frequency is 10 Hz for both cameras and 40 Hz for the LiDAR. The perception system was mounted and tested on a telescopic forklift, namely a Bobcat-TL470, as shown in Figure 2.

### 2.2. Sensor Positions on Heavy Machines

The LiDAR and the fisheye camera used in the proposed perception system are installed on a rigid platform at the front of the machine, and the camera is placed above the LiDAR, as shown in Figure 2.

**Figure 3:**
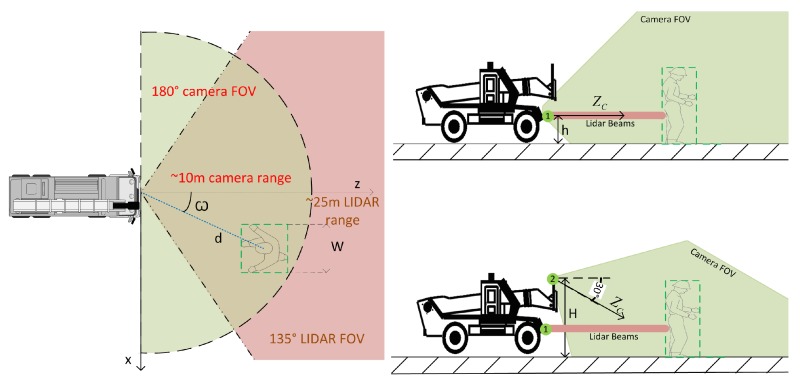
Sensor configuration map. Sensor heights are h=110cm and H=210cm, respectively.

The survey of accidents caused by heavy machines showed that the rear and the front are the most dangerous parts of a moving machine. Moreover, the areas around the machines are the most dangerous areas and are also blind areas for drivers. These findings indicate the usefulness of fisheye cameras mounted at the rear and front of the machines. Two positions for the fisheye camera can be defined and are denoted by Index 1 and 2 in Figure 3. Two different sensor configurations are then possible:

- Configuration 1: The two sensors are at Position 1. In this configuration, the sensors are kept at a low position (height h=110cm) and are usually parallel to the ground plane. This is the most commonly-used configuration in ADAS, because the appearance of the person on the image is essentially the same at any position in the FOV of the camera. This advantage is not preserved in fisheye images, since the appearance of the object is distorted, depending on its distance and its angle to the camera. A quantitative analysis of this distortion phenomenon is presented in Section 4.2.
- Configuration 2: The fisheye camera is mounted at Position 2 (height H=210cm, at the level of the forks), pointing down at an angle of exactly 30°. The LiDAR is at the same position as in Configuration 1. Cameras are usually placed high in heavy machines, so as to obtain a better coverage of blind angles around the machine and to avoid collisions that can damage the sensors. The case study reveals the additional advantage of showing the whole person at a very close range, even when physically touching the machine. The main drawback is that the fisheye transforms the shape of the person in a complex way.

In the same way, the multi-sensor system can also be mounted at the rear of the machine. With the same sensor height and angle, the sensor data from the front of the machine moving forward is equivalent to the data from the sensors mounted at the rear of the machine moving backward and *vice versa*. In practice, the position at the rear is more useful and makes maintenance easier. For safety reasons, the sensors are mounted at the front of the machine in all of the proposed data sequences.

### 2.3. The Heavy Machine Evaluation Dataset

The datasets have a very important role in the development of an object detection algorithm. A well-defined dataset is not only useful for evaluating a specific approach, but it also participates in the training process to improve the performance of the object detector. Most available datasets are either automobile-oriented or were built in the general context of pedestrian detection. One of the first and most widely-known datasets was made available in 2005 by Dalal *et al.* [17]. It consists of a large number of samples featuring people in a variety of postures and situations, obtained from photographs and from the Internet. Locations range from typical city streets to mountain landscapes. Between 2007 and 2009, a number of papers presented datasets to support new techniques. The Eidgenossische Technische Hochschule Zurich (ETH Zurich) dataset [18] was constituted to evaluate the approach known as “cognitive feedback”, which uses pedestrian location hypotheses together with depth cues to estimate the ground plane. Another dataset created at Technische Universitat Darmstadt (TUD) [19] was used to compare different image features and classification methods in real-time applications. The only available dataset in the context of heavy machines is the Human In work Machine Area (HIMA) dataset [20]) .

To the best of our knowledge, there are no other available datasets that provide both synchronized fisheye images and LiDAR data in the context of heavy machine environments. Moreover, this context has its own particular characteristics, including outdoor changing light conditions, strong vibrations from the engine and violent shocks that potentially make the detection process much harder.

To produce the dataset, the experiments are divided into seven scenarios for each configuration shown in Figure 2. The scenarios, defined within the experiments, aim to simulate situations frequently encountered on a construction site. People wore different kinds of clothes, including helmets, reflective vests and ordinary street clothes. Different situations of occlusions were also simulated.

The fisheye images are partially annotated and used for testing purposes. The annotations for the ground truth of these image sequences are done using the labeling tool of Dollár *et al.* [21]. Table 1 summarizes the main characteristics of the image sequences used in our tests.

**Table 1:** Test dataset statistics. Positive images each contain either one or more than one person, and each instance of people appearing in an image is considered as one positive sample.

Test Dataset	Number of Sequences	Positive Images	Positive Samples
**Configuration 1**	7	5747	13,045
**Configuration 2**	4	3570	9148

### 2.4. The Evaluation Method

Different metrics exist for evaluating the performances of an object detection framework. The evaluation method adopted in this work is used as the reference for the experimental results presented in the following sections. The usual procedure for evaluating binary classifiers is to select a training set and a testing set containing both positive and negative samples. A typical curve, such as the receiver operating characteristic (ROC) plots the false positive rate ($FPR=\frac{FP}{TN+FP}$) on the *x*-axis and the true positive rate ($TPR=\frac{TP}{TN+FP}$) on the *y*-axis using the number of true positives (TP), false negatives (FN), true negatives (TN) and the false positives (FP). In this paper, we followed the evaluation procedure of Dollár *et al.* [21] using their MATLAB evaluation toolbox [22]. In their work, the authors make use of a complementary curve, which plots the miss rate (MR=1-TPR) against the false positives per image (FPPI) on the *x*-axis. The curve of MR *versus* FPPI is obtained by varying the threshold of the detection confidence. Decreasing the threshold level reduces the miss rate and increases the false positives per image. In this case, lower curves indicate better performances of the detector. We use this curve throughout the paper, and we focus on the MR value when the FPR is in the range $10^{-2}$–$10^{0}$ FPPI. In the figure’s legend, the log-average miss rate is also used to summarize the detector performances. The log-average miss rate is computed by averaging the miss rate over nine FPPI rates evenly spaced in log-space in the range $10^{-2}$–$10^{0}$. When curves are essentially linear in this range, the log-average miss rate is similar to the performance at $10^{-1}$ FPPI [21]. The miss rate at $10^{-1}$ FPPI is used for curves that end before reaching the $10^{0}$ FPPI rate. The figure legend entries are ordered by log-average miss rate from the worst to the best (in percentage). During the evaluation, only bounding boxes with a height of more than 50 pixels are considered in the evaluation. Each detected bounding box may be matched once with the ground truth, and redundant detections are considered as FP.

## 3. Works Relating to Visual People Detection

Detecting people in images is a widely studied topic in research, and several survey papers [4,21,23,24] cover recent efforts and research programs dealing with the problem of pedestrian detection. We attempt to briefly present some important works, organized by different aspects: image features, learning algorithm and part-based approaches, to cover the main topics that we will address in this paper.

### 3.1. Image Features

The first measure taken to improve detection is to work on the image features. A variety of image features are used in recognition algorithms, but Haar and HOG (histograms of oriented gradients) features have received the most attention in the community. In [25], the authors introduced the Haar features used in the training of a classifier with a quadratic support vector machine (SVM). Later in [26], an approach was proposed using Haar-like features consisting of the original Haar features plus two similar features.

Dalal and Triggs [17] presented a human classification scheme that uses HOG features. It is noteworthy that HOG-based features have been extensively employed in the literature. Nearly all state-of-the-art PDSs use some derived version of HOG [4,21].

Additional cues often tend to have better performance in some restricted conditions. In [27], the authors emphasized the importance of depth and motion to improve pedestrian detection in an automobile context. In their work, multiple classifiers on intensity, depth and motion features are trained and combined using a neural network. Using depth and motion as additional cues in a component-based approach, they succeeded in reducing false positives by up to four times at a constant detection rate. In [28,29], the authors proposed combining HOG and local binary pattern (LBP) to effectively include texture information in the detection. In some works, HOG features with self-similarity features related to color channels are combined with motion features in order to better integrate spatial and temporal information [30]. In [31,32], the channel features are constructed by sum-pooling (filter bank convolution) over a set of rectangular regions, and the final feature vector is fed into a decision forest learned via AdaBoost.

While HOG (with its extensions) is undoubtedly the image feature most often used in people detection, it is not immediately obvious which are the best additional features. To the best of our knowledge, no methods have yet been proposed in this domain for successfully handling strong radial distortions from the camera optics. As a result and without loss of generality, we adopted HOG features as the principal features in our work. Through this paper, the name of *HOGreduced* stands for the HOG features proposed by [33] that are equivalent to the HOG features from [17] with a PCA-like dimension reduction to speed up the detection procedure.

### 3.2. Classification Algorithms

Classification algorithms have an important role in the PDS system. The PDS system receives a list of ROIs that are likely to contain a pedestrian, and using the features presented above classifies these ROIs as pedestrian or non-pedestrian, with the goal of minimizing the number of false positives and false negatives. The quality of the PDS is directly dependent on the quality of the learning algorithms employed, in terms of both detection performance and practical implementations.

Because of the real-time detection constraints, three classifiers are used almost exclusively. Support vector machines (SVMs) [34] help to find a decision boundary by maximizing the margin between the classes. SVMs were used in [35] with intensity images, in [25] with Haar features and in [17,33] with HOG. The AdaBoost algorithm [36] constructs a strong classifier by weighting and summing weak classifiers iteratively. Each new classifier focuses on misclassified instances. Boosting methods can be combined with any classifier to find the weak rules (e.g., with SVMs in [37]). These methods were used in [26,38] with Haar features and in [39,40] with HOG and shape context. Neural networks [41] use different layers of neurons to provide a nonlinear decision. Raw data are often used, *i.e.*, no explicit feature extraction process is needed. Raw data were used in [42] with intensity images, in [43] with gradient magnitude and in [44] with local receptive fields. Convolutional neural networks are also used as part of a deep learning approach for pedestrian detection [45]. These classifiers have different advantages and drawbacks, but they perform equally well in terms of detection performances in most of the benchmarks [21,24]. In people detection, there is no empirical evidence that one particular type of classifier performs better than the others.

Our concern in this paper is not modifying the classification algorithm itself, but adapting the training in the classification process to take distortions into account. The SVM classifier was used throughout our works because of its light computational load and its effectiveness. One other practical reason is related to the use of the deformable part model approach in our proposal, where the specific latent-SVM classifier was first used. The linear SVM can be considered as a special case of the latent-SVM classifier, where the latent variables are predefined. This means that we can use the same classifier tools for training and detection using the different detectors, which makes comparisons between detection algorithms more convenient, more objective and more accurate.

### 3.3. Part-Based Approaches

As stated above, single-model people detection approaches have their limitations when detecting people in different postures and with significant deformations of the image. A typical way of solving this issue is to use part-based approaches, which combine the classifications of different parts of the body instead of classifying the entire candidate as a single entity. In [46], the authors used a quadratic SVM to independently classify four human parts (head, legs, right arm and left arm). The classification results for these parts are combined using another linear SVM, where the data vector represents the presence or absence of parts in the designated area of the 128×64 pixels window. In [47], the authors used 13 overlapping parts. The training set is divided into nine clusters according to changes in posture and lighting conditions, resulting in 9×13=117 classifiers. The outputs of the classifiers are fed as weak rules to an AdaBoost classifier that sets the final classification rule. Dollár *et al.* [48] proposed multiple component learning using Haar, gradient magnitude and orientation features.

The part-based approach [33] introduced the deformable part model (DPM), which sums the classification score of the candidate regions of interest and different dynamic parts, taking into account the deformation cost. This idea gave rise to other DPM-based variant approaches, such as the multi-resolution DPM proposed in [49].

## 4. Distortion Analysis for Fisheye Images

Usually, geometrical distortions in images can be ignored if they are low. In most optical systems, geometrical distortions are not critical. Fisheye images, on the other hand, contain strong radial distortions that are visibly noticeable, and the performances of detection algorithms can be strongly affected. Below, we describe the fisheye camera measurement model and use it to quantify the impact of distortions on the performances of the people detection algorithm.

### 4.1. The Fisheye Camera Measurement Model

Most cameras can be modeled using a perspective imaging model where an optical system focuses light onto the image sensor. A perspective camera typically covers 45°–60° of the horizontal field of view. An important characteristic of perspective cameras is that they preserve the rectilinearity of the scene. This characteristic is very important and useful in many processes, including object recognition. Fisheye cameras can be modeled by perspective projection, but with noticeable geometrical distortions. Let ${}^{C}\tilde{P}={\left(X,\;Y,\;Z,\;1\right)}^{T}$ be the 3D position of a 3D homogeneous point in the camera frame C and $\tilde{x}={\left(x^{T},\;1\right)}^{T}$ its perspective projection in homogeneous coordinates on image I(x) at the point $x={\left(u,\;v\right)}^{T}$ (in pixels). The perspective projection is expressed as [50]:

(1) $$\lambda \tilde{x}=K[\begin{array}{ccc} I & | & 0 \end{array}]{\;}^{C}\tilde{P}$$

with:

(2) $$K=\left[\begin{array}{ccc} f_{x} & s & u_{0}\\ 0 & f_{y} & v_{0}\\ 0 & 0 & 1 \end{array}\right]$$

where $f_{x}$, $f_{y}$ are the focal length along each image axis, *s* is the skew factor (considered as zero in this paper) and $u_{0}$, $v_{0}$ are the coordinates of the principal point. When dealing with a fisheye camera, the pinhole projection coordinates u,v are replaced by the distorted coordinates $u_{d},v_{d}$ as follows:

(3) $$\left[\begin{array}{c} u_{d}\\ v_{d} \end{array}\right]=\left[\begin{array}{c} u\\ v \end{array}\right]+\overset{{\left(\delta_{u(x)},\delta_{v(x)}\right)}^{T}}{\overset{︷}{\underset{radial}{\left[\begin{array}{c} u_{c}\left(k_{1}r^{2}+k_{2}r^{4}+k_{3}r^{6}\right)\\ v_{c}\left(k_{1}r^{2}+k_{2}r^{4}+k_{3}r^{6}\right) \end{array}\right]}+\underset{tangential}{\left[\begin{array}{c} 2p_{1}u_{c}v_{c}+p_{2}\left(r^{2}+2{u_{c}}^{2}\right)\\ p_{1}\left(r^{2}+2{v_{c}}^{2}\right)+2p_{1}u_{c}v_{c} \end{array}\right]}}}$$

where $r^{2}={\left(u_{c}/f_{x}\right)}^{2}+{\left(v_{c}/f_{y}\right)}^{2}$ with $u_{c}=\left(u-u_{0}\right)$ and $v_{c}=\left(v-v_{0}\right)$. The geometrical parameters to be determined in the calibration process are the focal length $f_{x}$, $f_{y}$, the position of the principal point $x_{0}={\left(u_{0},\;v_{0}\right)}^{T}$ and the distortion coefficients $\left(k_{1},k_{2},k_{3},p_{1},p_{2}\right)$. To estimate these calibration parameters, we used the method proposed by [51].

### 4.2. Distortion Analysis of People’s Appearance

**Figure 4:**
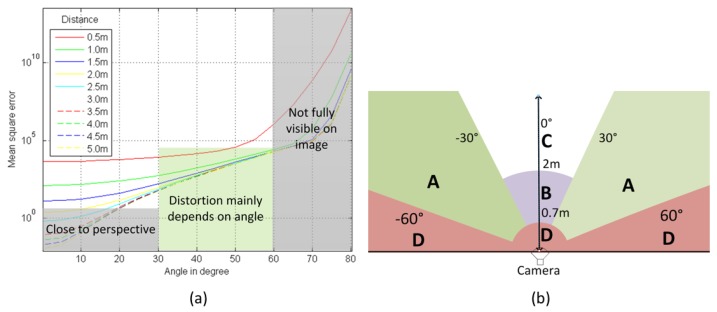
(**a**) $MSE_{p_{R}}$ *versus* the relative position of a person to the camera; (**b**) regions on the fisheye camera’s FOV.

The distortions in fisheye images are not uniform over all spatial areas, and this particularly affects detection at close range and at the image boundaries. The literature contains very little regarding the measurement of geometrical distortions in fisheye images and the impact of distortions on detection performance. In Figure 4, we illustrate these distortions using the relative positions of a hypothetical person and a fisheye camera. The flat-world assumption is used in order to convert a 3D rectangular region R of size W×H, parallel to the image plane and corresponding to a person in the 2D image space of size w×h. We assume that *H* = 1.7 m, and the width is defined as a ratio of the height W =*H*/2 = 0.85 m. This assumption has been widely used in the literature, mostly for automotive applications [10,52,53,54], and it implies that the person in the camera frame is on the ground in front of the vehicle and that the geometry of the road and its position with respect to the camera do not change. The center point ${}^{C}P_{R}$ of the rectangular region R is projected onto the image plane as $p_{R}$ using Equation (1). The distortion of an image point $p_{i}$, projected from the region R, is computed relatively to the center point as $p_{R}-p_{i}$. The mean square error ($MSE_{p_{R}}$) of all projection points is:

(4) $$MSE_{p_{R}}=\frac{\sum {\left(p_{R}-p_{i}\right)}^{2}}{w\;\cdot \;h}$$

Although the field of view FOV of a fisheye camera is up to 180°, a person is only fully visible on the image in certain areas. The boundaries of these areas are defined by the vertical and horizontal fields of view, VFOV and HFOV. If it is assumed that a person’s average height is *H* = 1.7 m, the minimum distance *d* of the person that is fully visible in the image can be computed as follows.

In our case, VFOV=108°, HFOV=144° and *d*= 0.62 m. The curves in Figure 4a show that the distortion of a person at a distance greater than 2 m can be ignored between 0° and 30°. The most interesting range to be considered is therefore between 30° and 60°, where the distortions depend very little on the depth distance and almost entirely on the radial angle. To restate this succinctly, we may say that the difference between fisheye and perspective images lies in Regions A and B (see Figure 4b). These areas are important in our system, because they correspond to the high-risk areas in heavy machines.

(5) $$d=\frac{H}{2\;\tan\;(\frac{VFOV}{2})}$$

### 4.3. Warping Fisheye Images

Given calibration information for a camera system, it is possible to remove distortions and to apply any image operator in a local perspective plane. Warping fisheye images into a local perspective image is the direct way to avoid non-perspective deformations. Warping an image at the VGA resolution (640×480) takes about 60ms (measured on a Windows 64-bit PC (CPU inter core i5 2.5GHz), by using the library OpenCV 2.4.), which cannot be ignored in real-time applications. Unfortunately, besides adding computational load, this approach has undesirable effects that we now discuss.

The non-uniform compression of the image structures (stretching effect) is a consequence of the wide-angle lens mechanism. More details are present at the center than at the edges of the image. In other words, the image sampling frequency decreases proportionally with the distance to the image center. The image rectification process warps the distorted wide-angle image into a local perspective plane. As a result, the boundaries of a rectified image will contain vacant pixels. These pixels are not directly mapped from the original image, but are often deduced from the neighbor pixels during an interpolation process [55,56]. The interpolation process in general has negative effects on image quality, which impacts the detection process, especially at the image boundaries. Danillidis [57] and Bulow [58] were among the first to argue that the warping of wide-angle images in general should be avoided in all image processing tasks. This conclusion was also supported by experimental results in the specific case of the Gaussian filter. Instead of warping images to a perspective plane, an alternative trend emerged: adapting classical image processing operations (such as filter, edge detection and SIFT) to spherical space when handling non-perspective images [59,60,61]. Later on, other researchers experimentally approved this conclusion [62,63] for segmentation processes on medical images.

## 5. The Mix Training Dataset Approach

Although HOG features and the sliding window paradigm obtain good detection performances, they are not designed to cope with strong distortions from fisheye images. The regular grid of the HOG-based feature, the rigid window and the regular step of the sliding window approach are not compatible with the irregular nature of the distortion fields in fisheye images.

In this section, we choose to continue using these two methods and focus on enhancing the training flow chart of the classification module. We assume that the supervised classifier can recognize pedestrians in distorted images when it has been trained with distorted sample images. The direct approach for solving the problem of distortion is to divide the field-of-view of the camera into sub-regions defined by angles and distances. To be able to apply this on different camera optics, distorted sample images are generated from perspective samples using a distortion simulation process. This kind of approach for object detection under different viewpoints using transformed samples to enhance the training datasets has been investigated in [64,65].

### 5.1. Simulation of Distortions

The proposed approach requires the training samples to be separated into subclasses depending on the degree of distortion. Wide-angle optics are very different from one to another, so the training dataset has to be adapted. Given that a significant amount of time and resources are required to build training and testing datasets, this represents a serious drawback. However, respecting the condition of genericity, it is possible to artificially deform existing perspective image datasets in order to simulate wide-angle camera images.

**Figure 5:**
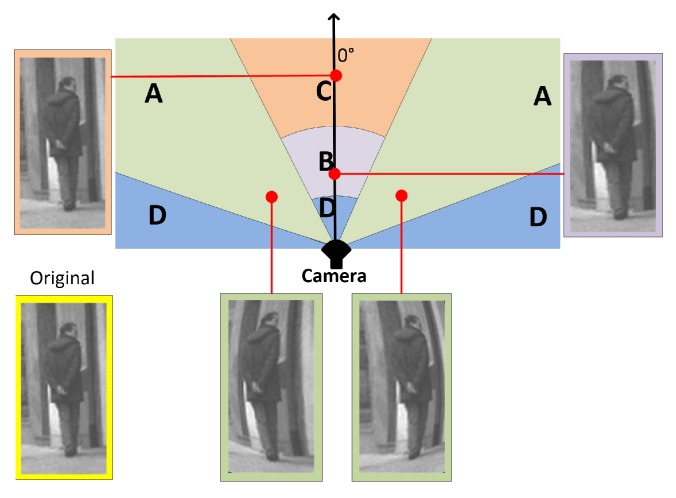
Artificial distorted images and their corresponding positions on the fisheye camera’s FOV. The sample image are simulated at -45°,0° and 45° at a distance of 1.2m.

Given an approximate size (W×H) of a person and its center position in the camera frame, a distorted figure can be computed using the camera projection equation (Equation (1)). Examples of simulated distortions are illustrated in Figure 5.

As proposed earlier, the bilinear interpolation method is used in all interpolation processes to fill in missing pixels. This degradation of image quality is correlated to the amount of distortion introduced, and it affects detector performance. In practice, the image samples lose most detail when the simulation angle is greater than 60°.

**Figure 6:**
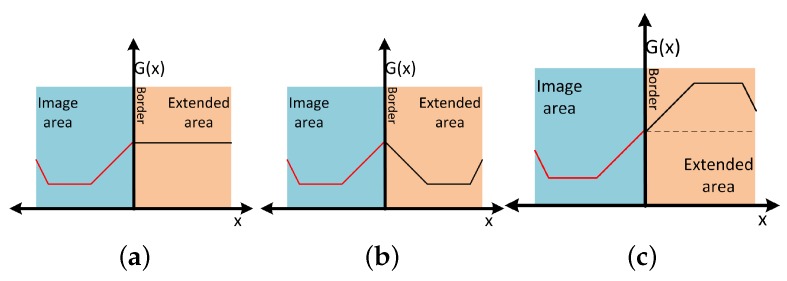
Examples of pixel values in the padding zone for one line of the image. (**a**) Duplicate; (**b**) mirror; (**c**) mirror-inverted.

It is worth mentioning that during the distortion process, we use padding pixels to widen the original sample so as to cover the warped areas around the distorted sample. The performance of the mix training dataset approach may be improved by optimizing this padding process. In many cases, the training dataset consisted only of examples of pedestrians of fixed size, without any global information about the scene. The image sizes of these datasets are relatively small, often at 48×96 or 64×128 pixels. Like the HOG approach in [17], the mix-training-dataset approach requires a wide background area around the person to get a good quality of distorted samples. The simulated image samples created by this process are not rectangular, like the original images. There exist black areas at the edges where no information from the original image is present. To solve this problem, we propose the frequently-used method of extending the border areas of the image sample. Different ways of replicating these pixels are available, and three of them are shown in Figure 6.

**Figure 7:**
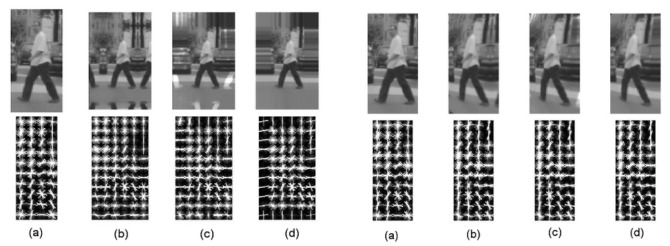
Visualization of HOG features from different border extension methods before (**left** part) and after (**right** part) the distortion process: (**a**) original image; (**b**) mirror extension; (**c**) mirror-inverted extension; (**d**) duplicate extension.

Figure 7 gives an example of different border extension functions before and after the distortion simulation. In the original images, the HOG features of different extension methods have noticeable differences. In the distorted version, on the other hand, few typical differences are to be seen between different extension methods. We observed the degradation of quality during the distortion simulation process, but this is not related to the padding area.

**Figure 8:**
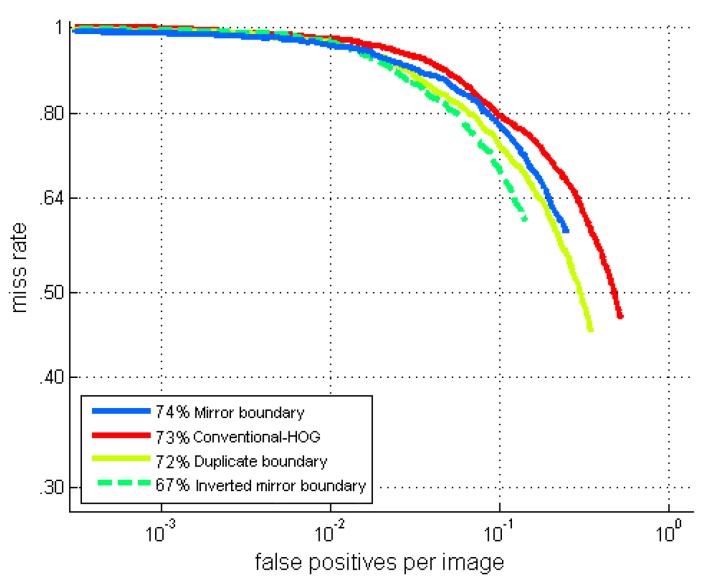
ROC curves of the mix-training-dataset approach with 50% distorted training dataset, using different padding functions.

In terms of the signal, the duplicate function gives strong contrast at the image borders, which is clearly visible on the gradient map. The mirror function often duplicates body parts in the padding image pixels. Only the inverted-mirror function gives more neutral border areas. It is therefore unsurprising that the inverted mirror method gives the best performances, as shown by the final results (see Figure 8).

### 5.2. People Detection Using the Mix Training Approach

Using distorted image samples generated by the method mentioned in the previous section, we propose a specific framework that we have termed the mix training approach for detecting people in fisheye images. To this end, the angles Θ=[-45°,0°,45°] and the distance *D* = 1.5 m were chosen to represent the critical areas A and B in the field of view.

**Figure 9:**
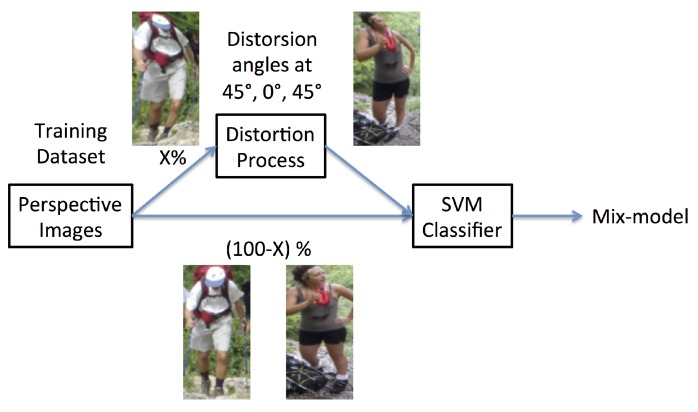
Flowchart of the proposed mix training detection approach.

In the mix training approach, only one classifier is used, just like in classical approaches (see Figure 9). The classifier is however trained on sample images without distortions, as well as on simulated distortions at different rates. Starting from a training dataset without distortions, we randomly replace a percentage of undistorted images by distorted ones. These distorted images are simulated at different distortion angles. Therefore, the total number of positive and negative sample images in the training dataset is the same in all cases. The percentage of distorted images in the training dataset can vary, and its effects on the performance of the detector are analyzed in the following section. At the final stage, a classifier merging mechanism is needed at the overlapping areas of the fisheye image. The winner-takes-all approach is adopted in our work for simplicity and computational reasons.

### 5.3. Experiments and Evaluations

**Figure 10:**
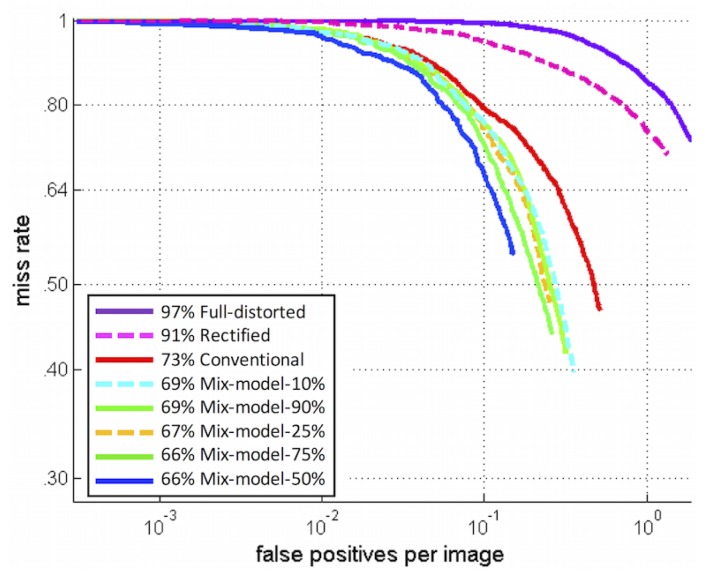
Results of different detectors trained with different percentage of distorted samples on fisheye test sequences.

The first experiment involves the classical HOG detector and the mix training dataset detectors at different percentages of distorted images (denoted mix model). The miss rate *versus* FPPI rate is plotted, and the log-average miss rate is used to summarize the detector performance. As specified in Section 2.4, the log-average miss rate is computed by averaging the miss rate over nine FPPI rates evenly spaced in log-space in the range $10^{-2}$–$10^{0}$. Figure 10 shows the full image evaluation of all the detectors, given the percentage of distorted images injected in the training dataset. In the figure’s legend, the notation “XX% Mix-model-YY%” means that the detector obtains XX% for the log-average miss rate at $10^{-1}$ FPPI and uses YY% of artificially-distorted samples in the training dataset.

The full distorted approach, which was trained with only distorted images, gives the worst performances. The degradation of image quality during the distortion process affects remarkably the performance of the detection. The rectified approach runs the detector on rectified fisheye images and shows poor detection results, as explained in Section 4.3. The conventional approach uses the Dalal *et al.* approach [17] directly on the fisheye images. Finally, the “XX% Mix-model-YY%” approaches gives the detector results using the proposed mix model. These results highlight that our mix training approach outperforms classical detection approaches. However, in this figure, the “Mix-model-50%” gives the best results due to the data bias in the test set. Indeed, when the detector is run several times on different test sets, the results can switch between “Mix-model-50%”, “Mix-model-75%” or “Mix-model-25%”. Despite theses results, such an approach has the advantage of being generic, as it can be adapted to all camera optics with known distortions. The detection performances are only held back by the trade-off between the image quality and the amount of distortions added to the training examples.

**Figure 11:**
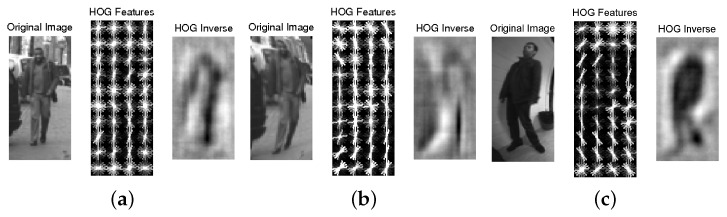
HOG features visualization in artificial distorted image samples. (**a**,**b**) The original 48×96 training sample image and its distorted version; (**c**) original distorted image sample captured by a fisheye camera.

In order to precisely analyze the effects of the distortion process, the HOG vectors may be visualized. In [66], the authors provided a trainable approach tool, HOG inverse, to reconstruct the original image from the HOG vector. Two examples of the distortion process applied to human figures are shown in Figure 11.

Figure 11c shows the same visualization of HOG features computed from an original distorted image. From a raw observation, it is not easy to find any significant differences. The HOG inverse results of the given artificial distorted samples give no hint of a human form (even for the perspective one). Head, shoulders and leg silhouettes are fading, in contrast to the original distorted fisheye sample. All of the details and sharpness of these samples are lost because of the artifacts produced by the interpolation step. Therefore, the HOG inverse approach can be used to select which samples have been properly distorted and which interpolation method to choose to obtain more reliable results.

## 6. The Adaptive Deformable Part Model

The experimental results described above show that modifying the training flow chart can lead to a reasonable improvement in detection performances in fisheye images. However, fitting the training samples to the model is not the best way of handling the deformation of human figures. It is hard to bypass the trade-off between the amount of distortion introduced in the sample images and the quality of those images. This goes back to the classic problem of interpolation. However, the problem can be solved differently, by adapting the detection model to the radial distortions using the DPM model.

### 6.1. The Deformable Part Model Approach

The idea of a deformable part model is to represent an object model using a “root” filter $f_{0}$ and a set of spatially-flexible higher-resolution “part” models $p_{i}=\left(f_{i},v_{i},d_{i}\right)$ [33]. Each part captures local appearance properties of an object with a part filter $f_{i}$, and the deformations are characterized by the deformation costs $d_{i}$. It seems intuitively obvious that the anchor position $v_{i}$ is the most favorable position of the *i*-th part with respect to the root position. From that anchor, the deformation cost is computed using a quadratic equation with four coefficients in $d_{i}={\left(x,y,x^{2},y^{2}\right)}^{T}$. An object hypothesis determines the location of each filter in the part-based model in a feature pyramid, $z={\left(m_{0},\ldots ,m_{n}\right)}^{T}$, where $m_{i}={\left(x_{i},y_{i},s_{i}\right)}^{T}$ specifies the position and scale of the *i*-th filter. The DPM approach employs the sliding window paradigm. The root filter $f_{0}$ and different part filters $f_{i}$ are applied all over the image to detect at the same time the whole body response and each part. In the part-based model, each sample $x={\left(I,m\right)}^{T}$ (denoting an image *I* in vector form and a position and scale m within the image) is given a score using a function of the form:

(6) $$g_{w}\left(x\right)=\max_{z}\;w\;\cdot \;\Phi \left(x,z\right)$$

The purpose of the training process in the latent SVM process is also to learn the vector of model parameters $w=(f_{0},z,b)$ from a set of labeled samples, and *b* is a real-valued bias term used to make the scores of multiple models comparable when combined into a mixture model.

Once the vector w has been obtained through training, the score of one object hypothesis can be computed as [33]:

(7) $$S_{H}\left(z\right)=\sum_{i=0}^{n}f_{i}\;\cdot \;\phi \left(I,m_{i}\right)-\sum_{i=1}^{n}d_{i}\;\cdot \;\psi \left(m_{i},m_{0}\right)+b$$

where $f_{i}.\phi \left(I,m_{i}\right)$ denotes the score obtained by applying filter $f_{i}$ at the position and scale specified by $m_{i}$. The vector $\psi \left(m_{i},m_{0}\right)={\left(dx_{i},dy_{i},dx_{i}^{2},dy_{i}^{2}\right)}^{T}$ with ${\left(dx_{i},dy_{i}\right)}^{T}={\left(x_{i},y_{i}\right)}^{T}-\left[2{\left(x_{0},y_{0}\right)}^{T}+v_{i}\right]$ gives the displacement of the *i*-th part relative to its anchor position $v_{i}$.

### 6.2. Adapting the DPM to Distortions

Two remarks may be made about the appearance of a person in fisheye images. First, the distortions are not uniform over all of the image area, but are more significant at close range and at the image boundaries. Second, the distortions of the different body components are minor compared to the distortion of the body as a whole. Starting from these observations and our understanding of the DPM (described in Section 6.1), we propose the following assumptions:

- Since the DPM approach has good performances when detecting people in perspective images, it should have equally good performances when detecting people at long distances in fisheye images.
- In extreme cases when people are very close and at the edges of the camera’s field of view, there will be a low response from the root filters $f_{0}$, but high responses from part-filters $f_{i}$. The deformation features $d_{i}$ and anchor positions $v_{i}$ may therefore be adjusted in the matching deformable cost function of the DPM approach (see Equation (7)) so as to adapt the deformable part model to the radial distortions. This adaptation will depend on the position of the object in the FOV of the camera. Once the anchor positions $v_{i}$ are well defined, the impact of distortions on the deformation features $d_{i}$ is therefore reduced.

We have developed the second assumption into an adaptive-DPM approach where the anchor positions of each part are relocated directly in the deformation model. Given a trained DPM model with parameters w, root location $m_{0}$ and anchor positions $v_{i}$, the anchor positions are transformed using the distortions model:

(8) $$v_{di}=v_{i}+{\left(\delta_{u(m_{i})},\delta_{v(m_{i})}\right)}^{T}$$

with ${\left(\delta_{u(m_{i})},\delta_{v(m_{i})}\right)}^{T}$, the distortion vector defined in Equation (3). Then, the score of one object hypothesis (Equation (7)) in the adaptive model will have ${\left(\tilde{dx_{i}},\tilde{dy_{i}}\right)}^{T}={\left(x_{i},y_{i}\right)}^{T}-\left[2{\left(x_{0},y_{0}\right)}^{T}+v_{di}\right]$. This gives the displacement of the *i*-th part relative to its anchor position using the fisheye distortion parameters.

**Figure 12:**
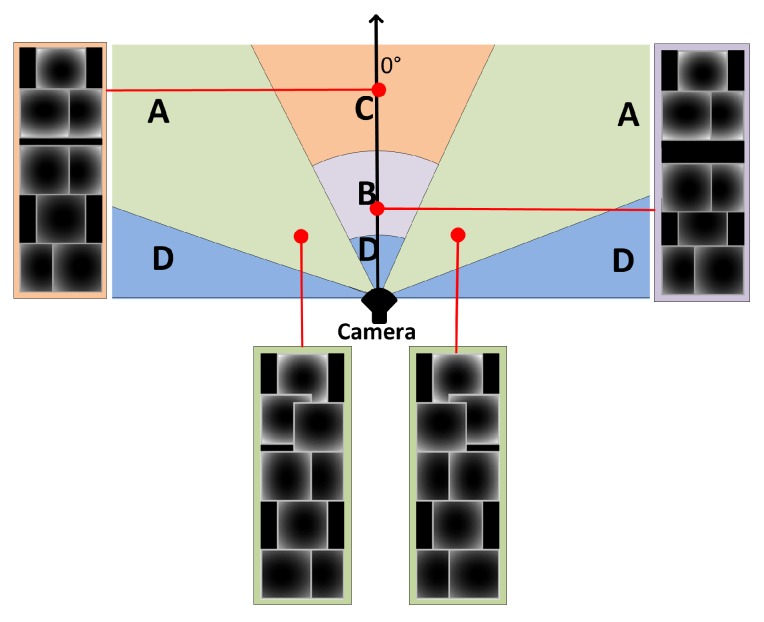
Illustration of adapted deformable part model to fisheye FOV.

As proposed in [33] and applying the distortion model, the detection in the fisheye image is done by accumulating a score for each root filter location $m_{0}$ according to the best possible positioning of all of the model parts:

(9) $$\begin{array}{ccc} S_{D}\left(m_{0}\right) & = & \max_{m_{1},\ldots ,m_{n}}S_{H}\left(z\right) \end{array}$$

(10) $$\begin{array}{ccc} & = & f_{0}\;\cdot \;\phi \left(I,m_{0}\right)+\sum_{i=1}^{n}\beta \left(m_{i},m_{0}\right)+b \end{array}$$

where:

(11) $$\beta \left(m_{i},m_{0}\right)=\max_{m_{i}}\;\left[f_{i}\;\cdot \;\phi \left(I,m_{i}\right)-d_{i}\;\cdot \;\Gamma \left(m_{i},m_{0}\right)\right]$$

with $\Gamma \left(m_{i},m_{0}\right)={\left(\tilde{dx_{i}},\tilde{dy_{i}},{\tilde{dx_{i}}}^{2},{\tilde{dy_{i}}}^{2}\right)}^{T}$, defines a generalized distance transform that spreads high filter scores of the part to nearby locations, by taking into account the deformation cost $d_{i}\;\cdot \;\Gamma \left(m_{i},m_{0}\right)$. The displacement of each part $v_{di}$ depends on the position of the person in the camera coordinate frame, and this displacement changes according to the position of the root filter location $m_{0}$ and the scale $s_{i}$ in the feature pyramid. Figure 12 illustrates the corresponding positions of the *i*-th part’s anchor of the model at different angles and distances to the camera. For each root position $m_{0}$ at a scale $s_{i}$, there is therefore one specific value of $v_{di}$, which can be computed offline using the camera calibration parameters and stored in a look-up table using Equations (3) and (8).

### 6.3. Influence of the Training Dataset

The first experiment involves the DPM approach trained with three different datasets, namely Inria, Inria-Mix and Fisheye Dataset (see Table 2).

**Table 2:** Training datasets’ characteristics.

Training Dataset	Positive Samples	Negative Samples
**Inria**	1371 samples from 614 images	1218 images
**Inria-Mix**	1371 samples from 614 images	1218 images
**Fisheye**	1520 samples from 810 images	1202 images

**Figure 13:**
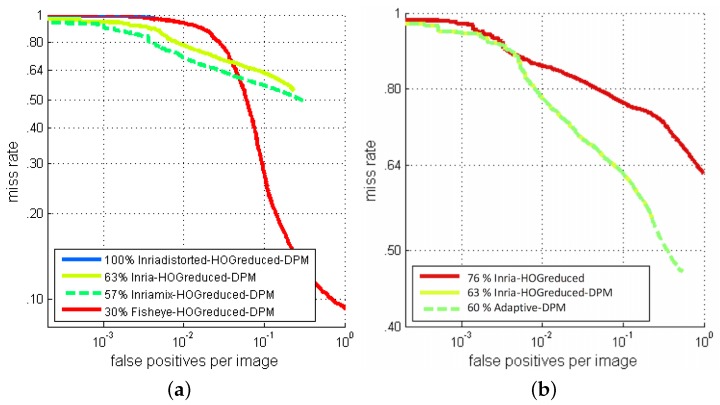
Detection performance of the deformable part model (DPM) approach trained with different datasets (**a**) and the adaptive-DPM approach (**b**).

The differences between the model trained with Inria and Inria-mix are not visually noticeable, but there are improvements in the performances (see Figure 13a). Enriching the training dataset can help to handle distortions of human figures, even in the DPM approach. As stated above, this process has the drawback of introducing missing pixels that are filled by interpolation. This phenomenon is proportional to the amount of distortions, and it has negative effects on the performance of the detector. The results of the Inria-mix detector in this experiment are the best that we can obtain from the mix training-dataset approach. The degradation of the image quality during the distortion process significantly affects the performance of the detection. The model trained with 100% of distorted samples gives an unrecognizable person model, and in the experiments, the detector fails to make any correct detections.

In the perspective camera model, the HOG responses are strong with respect to the human silhouette, especially for the head, shoulders and legs [67]. Because of this, the DPM defines the anchor position of parts in the high response region of the root filter. In the case of the fisheye detector model, although the contours of a person are recognizable, there is very little correlation between the root filters of the perspective and fisheye models. The learned spatial model with anchor positions defined by the root filter is therefore different. It is also important to note that the correlation between human body parts and the model parts is no longer clear.

It is worth mentioning that in the case of the fisheye detector, training and test images are extracted from the same dataset. The images were captured with the same camera and configuration. All of these conditions improve the performance of the fisheye detector.

### 6.4. The Adaptive-DPM Approach Detection Results

**Figure 14:**
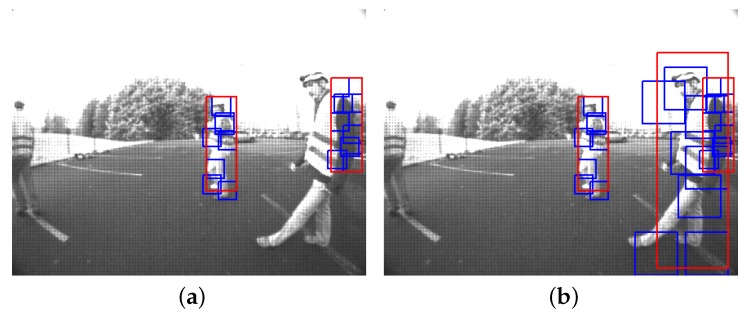
Examples of detection results with (**a**) the DPM approach and (**b**) the adaptive-DPM approach.

Here, instead of enriching the perspective training dataset by simulating fisheye distortions, we directly take into account the distortions in the detection model. The result shown in Figure 13b highlights the improvement in performance. Our proposed adaptive model has a clear advantage in extreme cases. In fact, even in the most extreme case, the dislocation of a part’s anchor ${\left(\delta_{u(x_{i})},\delta_{v(y_{i})}\right)}^{T}$ is smaller than 40% of the size of the root filter, while the distortion cost is calculated in the local region up to 80% of the size of the root filter around the anchor point. When the response of the part filter $f_{i}$ is high, the value will be propagated far enough to take the part contribution in the model. Relocating the anchor position is useful in difficult cases when a weak filter response appears. An illustrative example is shown in Figure 14.

## 7. Toward a Multi-Sensor People Detection System

Thanks to recent developments in sensing technologies, multi-sensor systems are becoming more and more accessible. Range sensors and cameras are now often used together, since they capture complementary information about the environment and can be used in collaboration for a more robust environment sensing system. Here, we propose a sequential single-layer LiDAR-based fusion architecture to improve the performance of the PDS and to handle different camera configurations.

### 7.1. Related Works

A system that detects, classifies and tracks a set of objects was proposed in [68]. Basic segmentation and classification methods were presented together with the linear discrete Kalman filter (KF). In [69], people are modeled as free-moving LiDAR segments that can be tracked by KF and classified using a majority-vote multi-hypothesis approach. In [70], the authors used a multi-model Gaussian mixture model (GMM) classifier on a set of laser-based features to recognize multiple object classes, include pedestrians. More recently, the LiDAR-based pedestrian detection presented in [9] was achieved by means of featureless approach.

The system proposed in [71] focused on solving practical issues in a LiDAR-based automotive application: the issues addressed include data association uncertainties and segmentation errors, as well as time processing and memory limitations. Other works in [72] attempt to estimate the ego-vehicle localization and speed of objects speed and to use this information in the classification of pedestrians. A set of features are used to model the pedestrian-likelihood. A maximum *a posteriori* (MAP) estimation is used as the classifier, where the prior was obtained using a supervised training process. In [73], people detection uses a multi-layer LiDAR. Their method uses a non-parametric kernel density-based estimation of pedestrian position of each laser plane. The results of the pedestrian estimations are then merged with respect to the four planes, after which the temporal filtering of each object is achieved via a stochastic recursive Bayesian framework (particle filter).

About a decade ago, a trend emerged that sought to combine 2D LiDAR with cameras in order to obtain better performances in people detection. In [74], the authors proposed using a LiDAR to define ROIs on image frames and to restrict the areas of detection. In [12], the authors used the range information provided by the LiDAR during the classification to deal with the problem of object scale variations in the images. The systems proposed in [10,11] use LiDAR to provide a list of ROIs in which a pedestrian may appear, while the camera is employed to classify potential pedestrians in the ROIs. In that work, a Bayesian framework is used as the fusion rule. In [75], the proposed approach merges LiDAR and camera data using a “decentralized approach”, where data from each sensor are processed independently, and the final decision is obtained by fusion rules.

### 7.2. LiDAR Objects

The LiDAR coordinate system origin point is defined as the laser emitting point. The raw LiDAR data is a 3D point cloud. A single 2D scan is a sequence of $N_{S}$ laser measurements $S=\{\left(\theta_{l},d_{l}\right)\;|\;l=1,\ldots ,\;N_{S}\}$ where $\left(\theta_{l},d_{l}\right)$ denotes the polar coordinates of the *l*-th scan point. These can be converted into Cartesian coordinate as $S=\{\left(d_{l}\cos\theta_{l},d_{l}\sin\theta_{l}\right)\;|\;l=1,...,N_{S}\}$ or ${}^{L}p={\left(d_{l}\cos\theta_{l},0,d_{l}\sin\theta_{l}\right)}^{T}$.

The goal of our system is to localize an obstacle and to classify it as a “person” or a “non-person”. The LiDAR data segmentation process is the first phase in obstacle detection. Raw LiDAR cloud points give dense range information in a 2D horizontal layer. A single 2D scan is a sequence of $N_{S}$ laser measurements $S=\{\left(\theta_{l},d_{l}\right)\;|\;l=1,\ldots ,\;N_{S}\}$, where $p_{l}=\left(\theta_{l},d_{l}\right)$ denotes the polar coordinates of the *l*-th scan point. This raw information is not necessarily convenient for determining the position of obstacles.

**Figure 15:**
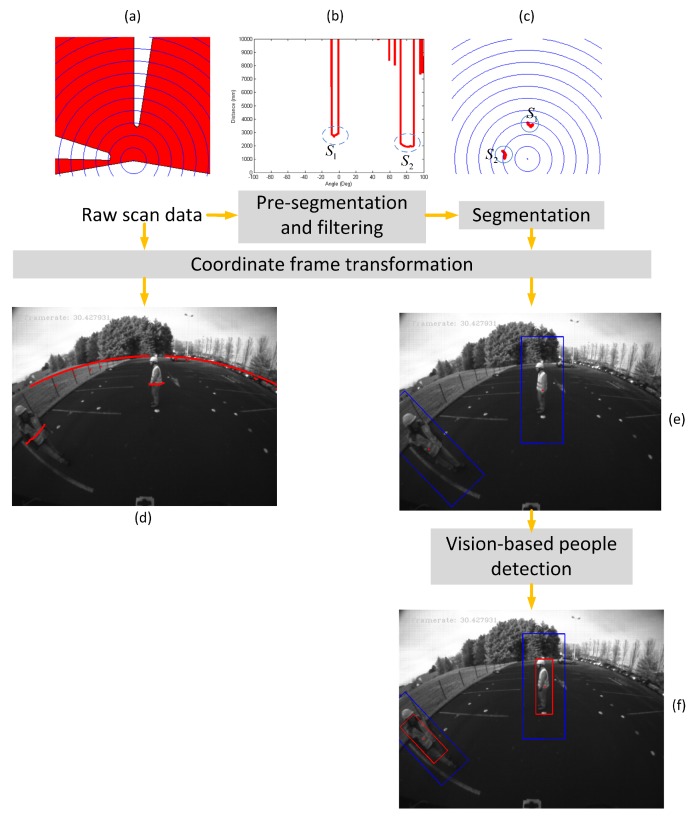
Processing steps in the LiDAR-based pedestrian detection system (PDS). (**a**) LiDAR points in polar coordinates; (**b**) point distance filtering; (**c**) two segments correspond to two obstacles in the FOV; (**d**) projection of points using calibration information; (**e**) image projection of adaptive ROIs; (**f**) final result of LiDAR-based PDS.

In Figure 15a,c, it can be seen that most of the LiDAR points are outside the distance of interest. An obstacle is characterized by a cluster of LiDAR points that we call a segment. A segment is composed of a set of laser points sharing similar spatial properties. A given segment is defined by the set $S_{k}:k<N_{S}$. The segmentation methods most often used for LiDAR data are distance-based methods. In this paper, we use the approach presented in [76], since we are using only a single-layer LiDAR. However, more advanced approaches, like the one presented in [77], can be used for multi-layer LiDAR.

As stated above, each obstacle is characterized by a cluster of LiDAR points. A cluster $S_{k}$ can be expressed as $S_{k}=\left\{\left(\theta_{m},d_{m}\right)\;|\;m=b_{k},...,b_{k}+n_{k}\right\}$, where $n_{k}$ and $b_{k}$ are respectively the number of points and the initial scan point of the cluster $S_{k}$.

Now, an obstacle is represented as a cluster $S_{k}$ in the LiDAR frame. Segments $S_{1}$ and $S_{2}$ extracted from a scan *S* are shown as an example in Figure 15a–c. We denote as $S:S_{k}\subseteq S$ the set of segments obtained by the segmentation stage. These segments will subsequently be used as inputs for the feature extraction and classification layer, as objects of interest.

In practice, we are interested only in the centroid point ${}^{L}C_{k}=\left(\theta_{C_{k}},d_{C_{k}}\right)$ and the size $D_{k}$ of the cluster $S_{k}$. Here, we assume that the size of a cluster is represented as the Euclidean distance between its endpoints:

(12) $$D_{k}=\sqrt{d_{b_{k}}^{2}+d_{b_{k}+n_{k}}^{2}+2.d_{b_{k}+n_{k}}.d_{b_{k}}\;\cos\;\left(\theta_{b_{k}+n_{k}}-\theta_{b_{k}}\right)}$$

The detection time of the system is increased as a result of the additional processing steps performed, namely the rejection of outliers, the detection of out-of-distance points and the determination of effective FOV.

### 7.3. Projection of Adaptive ROIs onto the Image Frame

The rigid body transformation between the LiDAR L and the camera C frames is denoted as ${}^{C}{\left[R,t\right]}_{L}$, where ${}^{C}R_{L}$ is the rotation matrix and ${}^{C}t_{L}$ is the translation vector. A point ${}^{L}p$ in the LiDAR frame is projected into the camera frame as:

(13) $${}^{C}p{=}^{C}R_{L}\;\cdot {\;}^{L}p{+}^{C}t_{L}$$

The extrinsic calibration parameters ${}^{C}{\left[R,t\right]}_{L}$ between the LiDAR frame and the camera frame are obtained through the calibration process presented in [78].

**Figure 16:**
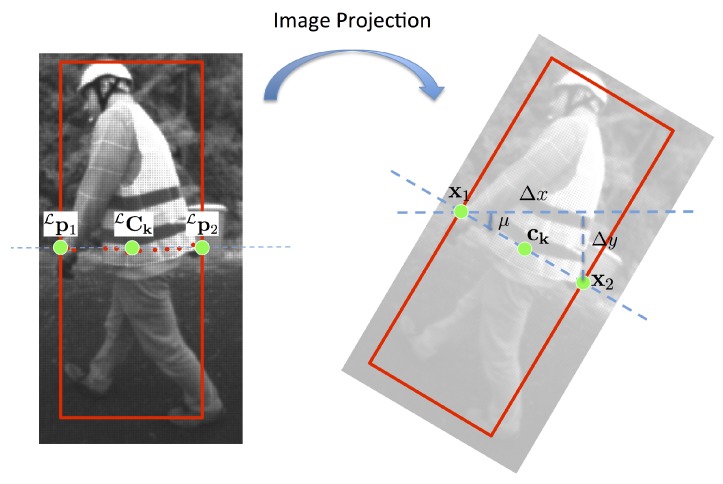
Computation of the ROI angle on the image.

For each detected object from the LiDAR data segmentation result, the three characteristic points ${(}^{L}C_{k},{\;}^{L}p_{1},{\;}^{L}p_{2})$ are expressed in the camera frame as ${(}^{C}C_{k},{\;}^{C}p_{1},{\;}^{C}p_{2})$ using the LiDAR-to-camera calibration parameters and then as $\left(c_{k},\;x_{1},\;x_{2}\right)$ on the fisheye image using the model of Equation (3). As shown in Figure 16, the angle *μ* between the obstacle appearance and the horizontal line in the fisheye image is then:

(14) $$\mu =\arctan\frac{\Delta y}{\Delta x}$$

where ${\left(\Delta x,\Delta y\right)}^{T}=\left|x_{2}-x_{1}\right|$. In our proposed approach, we do not use the geometric characteristic of the cluster as a cue to recognize the obstacle. Only the cluster size helps to determine the size of the ROI in the fisheye image. Therefore, for any configuration of the LiDAR/camera sensors, one LiDAR cluster defines one region of interest $ROI_{cam}=(c_{k},||x_{2}-x_{1}{\left|\right|}_{2},\mu )$ on the image. This is very convenient for an image-based people detection algorithm and makes the requirement of a rotation-invariant method unnecessary.

### 7.4. Experiments and Evaluations

In terms of computational cost, combining a LiDAR with a fisheye camera significantly improves the speed of the people detection system. While the complexity of the obstacle detection in the LiDAR data and in the ROI projection states is always the same, the speed of the people detection via the DPM approach depends directly on the area of the ROIs. We noticed a factor of about 20 for the detection time, measured using 300 images at VGA resolution on a desktop computer without any specific hardware acceleration. It is worth mentioning that the movements of heavy machines are normally much slower than automobiles, and a detection frequency of 10 Hz can be considered sufficient.

Below, we are more interested in comparing the detection performances of the proposed approaches. Different experiments were done using three detectors: the HOG proposed by [17], the adaptive-DPM approach and the LiDAR-DPM approach involving an adaptive-DPM approach and the segmentation of LiDAR data. The performances of the three detectors are evaluated from three standpoints: sensor configurations, person-to-machine distances and machine states.

#### 7.4.1. Sensor Configurations

Because the people detection algorithm is strongly variant to camera viewpoint, we chose to divide the dataset into two configurations, according to the dataset descriptions (Section 2.2).

##### Configuration 1

**Figure 17:**
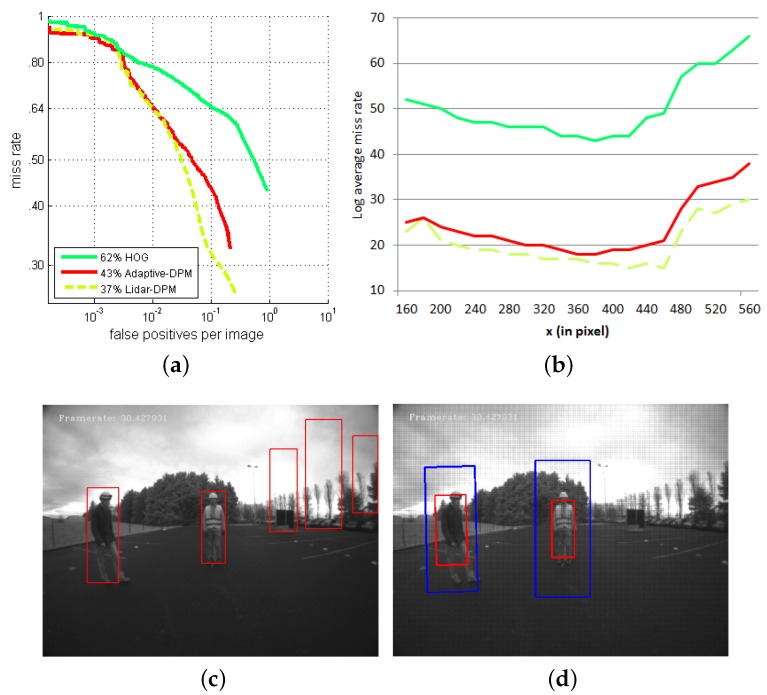
Comparison of detection performance between the exclusively vision-based approaches and the LiDAR-fisheye camera PDS in Configuration 1: (**a**) ROC curve, (**b**) miss rate for lateral image position, (**c**) vision-only detections and (**d**) LiDAR-fisheye detections.

The ROC curves in Figure 17a show that the adaptive-DPM approach performed better than the HOG detector in people detection on fisheye images because of the flexible model of the deformable parts. Introducing a LiDAR in a multi-sensor system is seen to improve detection results further by eliminating false detections in regions where an obstacle could not possibly be present.

Figure 17b shows the performances of all three detectors according to the horizontal position of a person in the fisheye images. The evaluation process is similar to what we presented in Section 5.3, where detection results are compared to the ground truth annotation on a region of (240×480) pixels. We wish to compare experimentally the effects of the distortion rate on the three people detectors by sliding this region horizontally across the image. As expected, the LiDAR-DPM detector outperforms the others at all angles. The improvement in performance obtained by integrating the LiDAR into the system is not obvious, which implies that the adaptive-DPM alone is already rather good at limiting the false detection rate.

The two images in Figure 17c,d illustrate a case where the LiDAR data help to eliminate false detections by the DPM detector. The difference in size of the result bounding box for the same person is apparent. This might be caused by the restriction of the ROIs, both in the background area and detection scale, by the multi-sensor detector.

The sliding window detection method requires multiple overlapping detections to be merged, given the requirement that a robust detector should give a strong positive response, even if the detection window is slightly off-center or off-scale on the object. As suggested in [17], we use non-maximum suppression when merging overlapping detections. The final bounding box result depends on multiple primary overlapped bounding boxes as the output of the classification. The merging method takes into account the detection scores, overlapping areas and the relative scale. We note that the positive responses of the detector are spread over an area that is larger than the ROIs defined by the LiDAR. As a result, the final bounding box of the adaptive-DPM detector is larger than the bounding box given by the merging system.

##### Configuration 2

**Figure 18:**
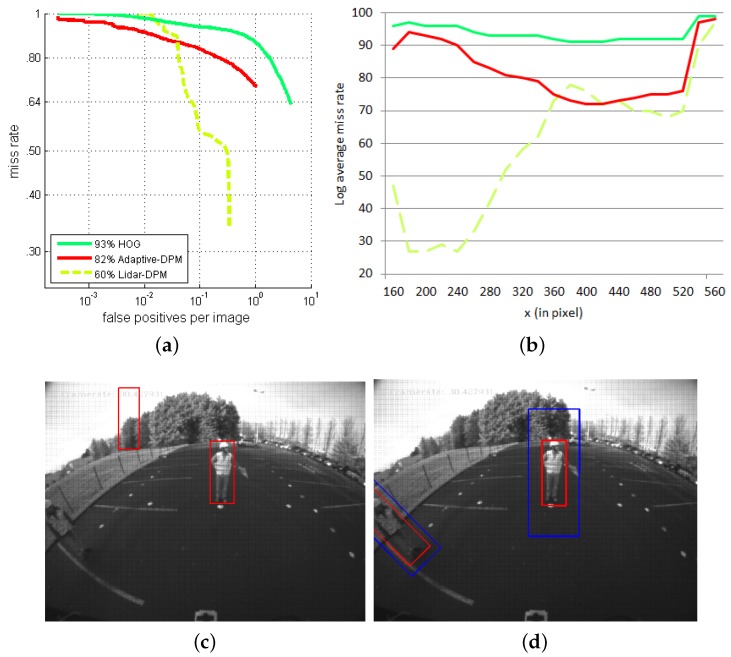
Comparison of detection performance between the exclusively vision-based approaches and the LiDAR-fisheye camera PDS in Configuration 2: (**a**) ROC curve, (**b**) Miss rate for lateral image position, (**c**) vision-only detections and (**d**) LiDAR-fisheye detections.

Figure 18a shows the performances of the LiDAR-camera system compared to the camera-only approach in Configuration 2. People on the sides of the sensors are both strongly distorted and rotated. Vision-based people detection methods that do not take these deformations into account give very poor performances. The system’s miss rate reaches 95% over the range of FPPI between $10^{-2}$ and 1, which we may consider a total failure. The LiDAR ensures that the ROIs corresponding to obstacles are rotated before the visual recognition is launched. In this favorable case, the DPM approach performs considerably better.

We can see this effect even more clearly in the log-average of miss rate curves along the horizontal axis in Figure 18b. The detection performance of the LiDAR-DPM detector is especially good on the left-hand side of the images. Figure 18c,d, extracted from the heavy machine dataset, captures the advantages of the LiDAR-camera PDS. While the vision-only approach cannot detect a rotated person in the fisheye image, the location information provided by the LiDAR can help to correct this rotation before detecting the person inside the ROIs.

The evaluation along the *x*-axis in Figure 18b has an interesting characteristic around x=400 pixels, which is near the image center. Here, the multi-sensor detector performs less well than the adaptive-DPM detector, even though they both use the same vision-based people detection algorithm. The explanation for this degradation must lie in the region of interest. Our opinion is that the background area around the person may not be large enough and that the ROIs should be enlarged to ensure the proper functioning of the detection algorithm.

#### 7.4.2. Machine States

**Figure 19:**
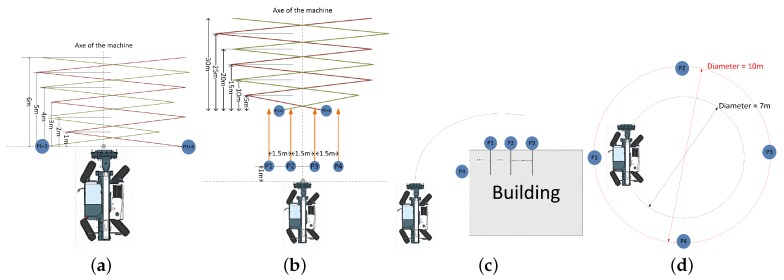
Diagram of the scenarios from the heavy machine dataset: machine stationary (**a**); machine moving forward (**b**); and machine turning (**c**,**d**).

From the same detection results, we evaluated the performances of the three detectors with respect to another criterion, namely machine state. The results are separated according to three possible operating states of the machine: stationary, moving forward and turning. These operating states are illustrated by the scenarios diagram used in our experiments (see Figure 19).

**Figure 20:**
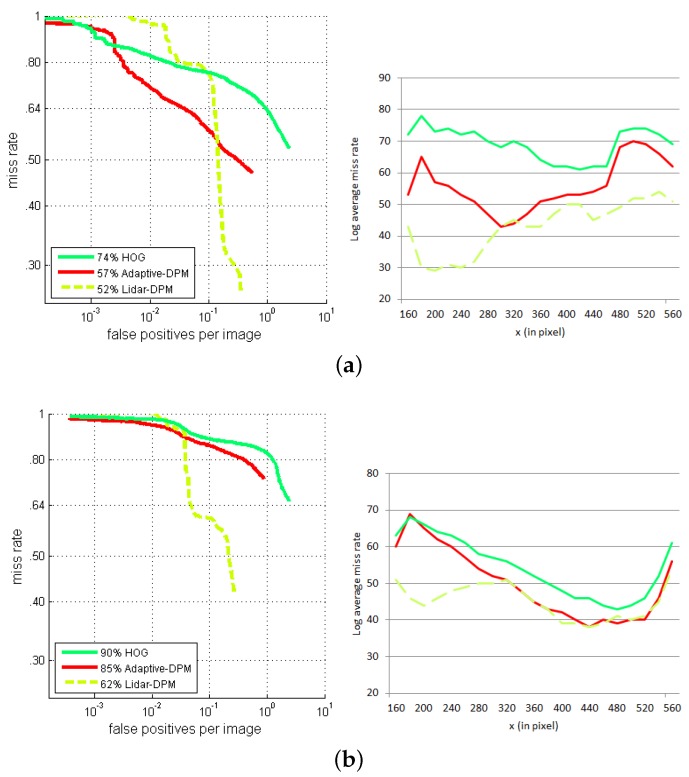

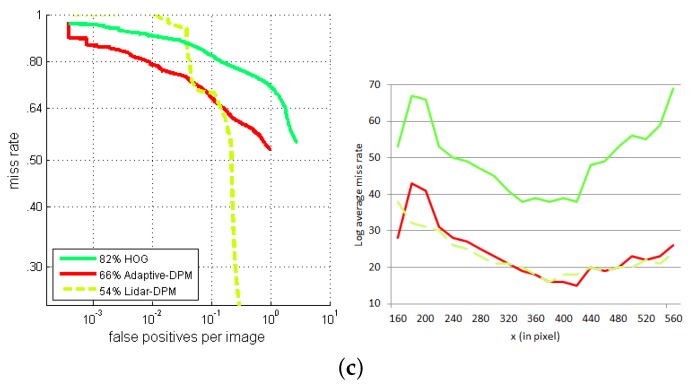
Comparison of detection performance between the exclusively vision-based approaches and the LiDAR-fisheye camera PDS in different machine operating states: (**a**) static, (**b**) turning and (**c**) forward.

Figure 20a shows the evaluation results when the machine is stationary, which corresponds to Scenarios 1 and 2 in the heavy machine dataset. Similarly, the evaluation results of the machine moving forward (Scenario 3) and moving in a circle (Scenario 5) are shown in Figure 20b,c. These results are in conformity with the overall conclusions: the performances of all three detectors are always better at the image center than at the edges. Moreover, the adaptive-DPM detector gives results that are always better than those given by the HOG detector, but worse than those given by the multi-sensor detector.

There are some exceptions where the adaptive-DPM works better than the multi-sensor detector. Such exceptions occur in all three scenarios, but always near the image center. We conclude that this phenomenon is not related to the operating state of the machine. It might be related to the size of ROIs projected on the image.

## 8. Conclusions and Future Work

People detection systems are undoubtedly the key technology for reducing the number of accidents caused by heavy machines in construction environments. People detection is by no means an easy task, given the difficulties that such systems encounter: the deformation of people’s appearance, real-time detection of moving targets in uncontrolled outdoor scenarios, sensor positions, *etc*. The main contributions of this paper are state-of-the-art people detection approaches and the proposal of an adaption for the context of heavy machines.

In the light of our analysis of the context of heavy machines, the perception system we chose consists of a monocular fisheye camera and a single-layer LiDAR. The great advantage of the fisheye camera in a PDS is that its very wide field-of-view covers the machine’s blind areas. In this paper, we have investigated and quantified the performance of this perception system, both theoretically and experimentally. Our proposal, based on state-of-the-art people detection approaches, was inspired in particular by the histogram of oriented gradient and the deformable part model. We have shown that our proposed perception system improves on the performance of a vision-based PDS system in fisheye images.

To handle the fisheye distortions, we proposed modifying the training and detecting flowcharts by including artificially-distorted samples into the training dataset. It was demonstrated that enriching the training dataset can help to handle the distortions of people’s appearance. However, fitting the training samples to the model is not the optimal way of handling deformations of people’s appearance, because it is hard to bypass the trade-off between the amount of distortion introduced in the sample images and quality of these images. These limitations can be overcome by adapting the DPM approach. The deformable models can be modified to be even better adapted to the strong distortions of the fisheye images. Such an approach has the drawback of high computational cost and complexity. For this reason, we have proposed a sequential LiDAR-based fusion architecture to directly address the problem of reducing false detections and computational cost in the exclusively vision-based system.

For the experimental part, a specific dataset was built. It was divided into two configurations. In the first configuration, the sensors are kept at a low position and are mostly parallel to the ground plane. This is the most commonly-used configuration in ADAS. In the second configuration, the downward-pointing fisheye camera is mounted at a high position. The high position of the camera is convenient for heavy machines because collisions that can damage the sensors are avoided, and it is possible to obtain a better coverage of blind angles around the machine. The results are promising, both in terms of processing speed and performance. This is especially true for the second configuration, where the rate of misdetections at $10^{-2}$ FPPI was less than 30%.

As extensions of the current work, we plan first to devote some effort to real-time optimizations using hardware-based solutions with parallelization of the DPM approaches. We are also currently working on the extension of a deep-learning-based approach for fisheye images [45] using both pixels and local spherical descriptors [79]. A specific heavy machine dataset is also under preparation to handle different postures of people working on construction sites. This topic can also be addressed using a deep learning approach, as in [80].
